# Prevalence of *ESR1* E380Q mutation in tumor tissue and plasma from Japanese breast cancer patients

**DOI:** 10.1186/s12885-017-3779-2

**Published:** 2017-11-22

**Authors:** Takashi Takeshita, Yutaka Yamamoto, Mutsuko Yamamoto-Ibusuki, Aiko Sueta, Mai Tomiguchi, Keiichi Murakami, Yoko Omoto, Hirotaka Iwase

**Affiliations:** 1 Department of Breast and Endocrine Surgery, Graduate School of Medical Sciences, Kumamoto University, 1-1-1 Honjo, Chuo-ku, Kumamoto City, 860-8556 Japan; 2 Department of Molecular-Targeting Therapy for Breast Cancer, Kumamoto University Hospital, 1-1-1 Honjo, Chuo-ku, Kumamoto, 860-8556 Japan; 3 Department of Endocrinological and Breast Surgery, Graduate School of Medical Science, Kyoto Prefectural University of Medicine, 465 Kajii-cho, Hirokoji Agaru, Kawaramachi-dori, Kamigyo-ku, Kyoto, 602-0841 Japan

**Keywords:** Metastatic breast cancer, Acquired endocrine therapy resistance, *ESR1* mutations, E380Q mutation, Cell-free DNA

## Abstract

**Background:**

*ESR1* mutations have attracted attention as a potentially important marker and treatment target in endocrine therapy-resistant breast cancer patients. The E380Q mutation, which is one of the *ESR1* mutations, is associated with estradiol (E2) hypersensitivity, increased DNA binding to the estrogen response element, and E2-independent constitutive trans-activation activity, but its frequency in *ESR1* mutations remains unknown. The present study aimed to investigate the E380Q mutation in comparison with the other representative *ESR1* mutations.

**Methods:**

We screened a total of 62 patients (66 tumor tissues and 69 plasma cell-free DNA (cfDNA)) to detect *ESR1* mutations (E380Q, Y537S, Y537N, Y537C, and D538G) using droplet-digital polymerase chain reaction. Plasma was collected at more than two points of the clinical course, in whom changes of *ESR1* mutations under treatment were investigated.

**Results:**

We detected *ESR1* mutations in 21% (12/57) of MBCs. The E380Q *ESR1* mutation was found in 16% (2/12) and the other *ESR1* LBD mutations were five (41.6%) of Y537S, and four each (33.3%) of D538G, Y537N, and Y537C, in 12 *ESR1* mutant breast cancer patients. Five tumors had multiple *ESR1* mutations: three had double *ESR1* mutations; Y537S/E380Q, Y37S/Y537C, and Y537S/D538G, and two had triple *ESR1* mutations; Y537S/Y537N/D538G. In plasma cfDNA analysis, the E380Q mutation was not detected, but increases in other *ESR1* mutations were detected in 46.2% (6/13) of MBC patients under treatment.

**Conclusions:**

We have shown that there are distinct populations of *ESR1* mutations in metastatic tissue and plasma. Each *ESR1* mutation may have different clinical significance, and it will be necessary to investigate them all.

**Electronic supplementary material:**

The online version of this article (doi: 10.1186/s12885-017-3779-2) contains supplementary material, which is available to authorized users.

## Background

*ESR1* ligand-binding domain (LBD) mutations that induce endocrine therapy (ET) resistance in breast cancer (BC) were first reported almost two decades ago [1–4] and novel developments of sensitive technologies such as next-generation sequencing (NGS) confirmed that *ESR1* LBD mutations act as drivers of ET resistance [5–8]. In addition, recent developments of digital genomic technologies revealed that plasma cell-free DNA (cfDNA) is an useful source to quickly assess the mutational profiles and monitor the molecular changes under treatment [9]. It has been reported the clinical significance of monitoring *ESR1* LBD mutations in plasma cfDNA [10–12]. In the BOLERO-2 study, Chandarlapaty and colleagues found that a total of 155 (28.8%) of 541 ER-positive MBC patients had the D538G and/ or the Y537S *ESR1* mutation in plasma cfDNA, which are the representative *ESR1* LBD mutations, and each of them was associated with shorter overall survival [13]. Interestingly, they also demonstrated an additional benefit of the use of mTOR (mammalian target of rapamycin) inhibitor depending on the *ESR1* LBD mutation present; the D538G *ESR1* mutation derived a large benefit from treatment with an mTOR inhibitor, whereas those with the Y537S mutation did not. These data suggest that each *ESR1* LBD mutation may play a different role and more work is needed to confirm this.

Pakdel and colleagues [14] found that mutation of the one charged amino acid, E380Q*,* resulted in a requirement for less estradiol than wild-type (WT) *ESR1* to achieve maximal activity and this mutation also showed high trans-activation activity in the absence of added hormone. They suggested that this *ESR1* LBD mutation may be important in DNA binding and protein–protein interactions that modulate transcriptional activity of the estrogen receptor (ER). After 20 years, the presence of the E380Q *ESR1* mutation came to be reported in both tumor tissue DNA (ttDNA) and plasma cfDNA [10, 14–19]. In the recent phase 2 clinical trial for plasma cfDNA of aromatase inhibitor (AI) resistant metastatic BC (MBC) patients, this mutation was found in 26% (15/57) of *ESR1* mutant plasma samples [18]. Fribbens and colleagues reported that the E380Q *ESR1* mutation was found in 9.5% (6/63) in the SoFEA study for hormone receptor (HR)-positive BC patients who had demonstrated prior sensitivity to AIs, but it was found in 24.4% (22/91) in the PALOMA3 trial for HR–positive BC patients who had progressed during prior ET [19]. These results suggest that E380Q *ESR1* mutation may be a marker for screen of ET-resistant BC like the other representative *ESR1* LBD mutations (Y537S, Y537N, Y537C, and D538G) [10–12, 20]. However, the literature contains little information regarding the E380Q *ESR1* mutation in Japanese BC patients. Thus, the present study screened for the presence of the *ESR1* E380Q mutation in ttDNA and plasma cfDNA of 62 ER-positive Japanese BC patients using droplet digital polymerase chain reaction (ddPCR) and compared the frequency with the representative *ESR1* LBD mutations (Y537S, Y537N, Y537C, and D538G). To our knowledge, this is the only precise study to use ddPCR to examine the presence of the E380Q *ESR1* mutations in a series of tumor tissue and plasma samples of Japanese BC patients.

## Methods

### Patients and breast cancer samples

A total of 62 patients (9 tumor tissue and 18 plasma samples in the primary BC (PBC) group and 57 tumor tissue and 51 plasma samples in the MBC group), treated at Kumamoto University Hospital between 2005 and 2014, were enrolled in this study. Informed consent was obtained from all patients before biopsy or surgery. The Ethics Committee of Kumamoto University Graduate School of Medicine (Kumamoto, Japan) approved the study protocol. The treatment for PBC was carried out in accordance with the recommendations of the St. Gallen international expert consensus on the primary therapy of early BC at that time and the treatment for MBC was carried out in accordance with the National Comprehensive Cancer Network Clinical Practice Guidelines in Oncology [21]. Patients were examined at the Kumamoto University Hospital or affiliated hospitals and were assessed for the presence or absence of relapse and for clinical response, which was defined according to the Response Evaluation Criteria in Solid Tumors as complete response, partial response, stable disease, or progressive disease during the follow-up period as described previously [22].

### Sample preparation

Tumor cells and plasma were processed and each solution, which was examined for quality and quantity, was used as template ttDNA and cfDNA for the analysis of *ESR1* mutations, respectively, as described elsewhere [22].

### Analysis of *ESR1* mutations by ddPCR

ddPCR assay was performed on the same sample twice using the QX200^™^ Droplet Digital^™^ PCR System (Bio-Rad Laboratories, Hercules, CA, USA) and PCR data were quantified using QuantaSoft^™^ software (Bio-Rad Laboratories) and results are expressed as fractional abundance (mutant allele frequency: MAF) for each tumor tissue sample and as copies/μL of mutant DNA for each plasma sample as described previously [22]. Our ddPCR analysis of four representative *ESR1* LBD mutations (Y537S, Y537N, Y537C, and D538G) had already been optimized by comparative analysis of a dilution series of each synthetic *ESR1* LBD mutant oligonucleotide as reported previously [20]. All samples were compared with the *ESR1* WT molecule and each *ESR1* mutant molecule as positive control. A water-only (no template) control and WT normal human DNA (*Taq*Man Control Genomic DNA) were run in parallel for each ddPCR reaction as negative control.

### Probes and primers

We used LBx^®^ Probe ESR1 E380Q (#65116) as the detection probe for *ESR1* E380Q (Riken Genesis, Tokyo, Japan) and Custom *Taq*Man SNP Genotyping assays (Applied Biosystems, Foster City, CA, USA) for the detection of other *ESR1* LBD mutations (Y537S, Y537N, Y537C, and D538G), as described previously [20].

### Site-directed mutagenesis

The gene art site-directed mutagenesis system (Life Technologies) was used to generate mutations within *ESR1* LBD. We generated *ESR1* E380Q using PrimeSTAR® GXL DNA polymerase (Takara Bio Inc., Shiga, Japan). WT ER expression vector (pcDNA-ER) was used as a template with the following mutagenesis primers:

E380Q: Forward, 5′-CTTCTACAATGTGCCTGGCTAGAGATCCT-3′’

Reverse, 5′-TACTAGTCCAGGTGGAAGATGTTACACGGA -3′’

The *ESR1* mutant molecule for ddPCR was amplified using PCR based on the *ESR1* mutated plasmid, as described [20].

### Immunohistochemistry

Immunohistochemical staining for ER alpha, progesterone receptor (PgR), human epidermal growth factor receptor 2 (HER2), and Ki67 was carried out on selected 4-μm thick tumor section. Primary antibodies, their visualization methods, and their evaluation were as previously described [23].

## Results

### Patient characteristics

**Fig. 1:**
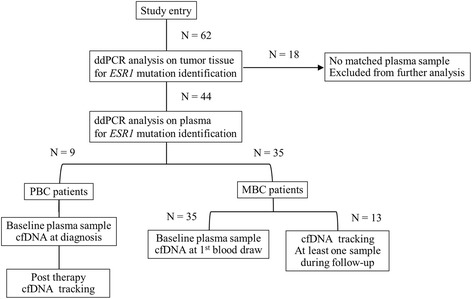
Schematic presentation of the protocol used in the present study. Abbreviations; ddPCR, droplet digital polymerase chain reaction; PBC, primary breast cancer; MBC, recurrent metastatic breast cancer; cfDNA, cell-free DNA

A total of nine (14.5%) of 62 women with PBC and a total of 53 (85.5%) of 62 women with MBC were enrolled (Fig. 1). In the MBC patient group, four women were biopsied in heterochrony twice and 57 BC specimens were evaluated for *ESR1* mutation analysis. Baseline characteristics of primary and metastatic ER-positive BC specimens are summarized in Table 1. The median age of the patients at tumor tissue biopsy was 56 years (range, 31–68) in the PBC group and 58 years (range, 31–95) in the MBC group. In the primary clinical stage, eight patients (14%) were categorized as stage III and ten (17.5%) as stage IV in the MBC group. Four representative *ESR1* LBD mutations (Y537S, Y537N, Y537C, and D538G) were previously evaluated with all samples of this study [12, 20]. The median number of metastatic organs was two (range, 1–5) and 49.1% of the patients had visceral metastasis. The median number of prior ET and prior chemotherapy treatments was two (range, 0–6) and 0 (range, 0–6), respectively. The median duration of follow-up was 77 months (range, 25–113) in the PBC group and 97 months (range, 4–290 months) in the MBC group.

**Table 1:** Patient characteristics

	No. of patients (%)	
	PBC	MBC
Variables	(*N* = 9)	(*N* = 57)
Age at biopsy		
Median (range)	56 (31–68)	58 (31–95)
Primary clinical stage		
I	2 (22.2)	22 (38.6)
II	6 (66.7)	17 (29.8)
III	1 (11.1)	8 (14)
IV	0	10 (17.5)
Histological type		
Invasive ductal	9 (100)	53 (93)
Invasive lobular	0	3 (5.3)
Mucinous	0	1 (1.8)
Histological grade		
1	6 (66.7)	10 (17.5)
2	2 (22.2)	27 (47.4)
3	1 (11.1)	17 (29.8)
Lobular	0	3 (5.2)
Median percentage of ERα (25%, 75%)	90 (75, 92.5)	85 (70, 90)
Median percentage of PgR (25%, 75%)	10 (5, 45)	30 (5, 60)
HER2		
Positive	0	5 (8.7)
Negative	9 (100)	52 (91.2)
Presence of *ESR1* LBD mutations		
Yes	0	11 (19.3)
No	9 (100)	46 (80.7)
Number of metastatic organs		
Median (range)		2 (1–5)
Visceral involvement		
Yes		28 (49.1)
No		29 (50.9)
Metastatic lesions biopsied		
Breast		9 (15.8)
Skin		19 (33.3)
Lymph Nodes		14 (24.6)
Bone		4 (7.0)
Lung		3 (5.2)
Liver		4 (7.0)
Brain		3 (5.2)
Ovary		1 (1.8)
Number of rounds of prior endocrine therapy		
Median (range)		2 (0–6)
Number of rounds of prior chemotherapy		
Median (range)		0 (0–6)

Abbreviations: *ERα* estrogen receptor alpha, *PgR* progesterone receptor, *HER2* human epidermal growth factor receptor 2, *LBD* ligand binding domain, *AI* aromatase inhibitor, *SERM* selective estrogen receptor modulator, *IBTR* ipsilateral breast cancer recurrence

### Quantitative analysis of ddPCR using the *ESR1* E380Q mutant molecule

**Fig. 2:**
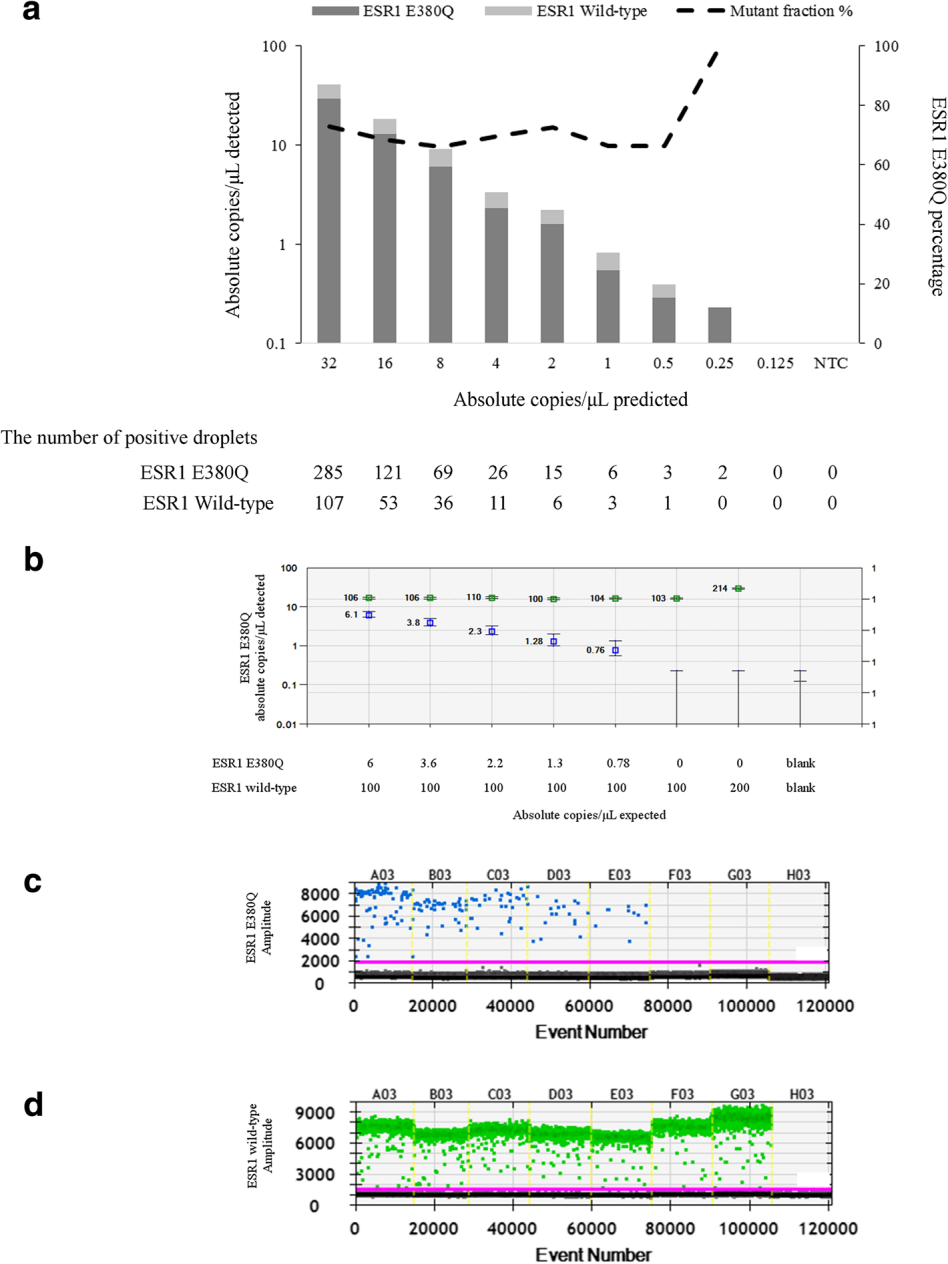
**a** Comparative ddPCR analysis of a dilution series of the indicated synthetic *ESR1* E380Q oligonucleotide. We used serial dilutions of the *ESR1* E380Q molecule and analyzed them by ddPCR. A mixture of recombinant *ESR1* E380Q and WT was plotted against the different fractional concentrations from 32 to 0 copies/μL. The MAF of *ESR1* E380Q was maintained at more than two droplets. Therefore, a mutation was only considered to be present if more than two positive droplets were detected. Abbreviations; ddPCR, droplet digital polymerase chain reaction; WT, wild-type; MAF, mutant allele frequency. **b-d** Dilution experiments where *ESR1* E380Q oligonucleotide was diluted in a background of WT normal human DNA are shown. The dilution experiments were prepared by 1.3-fold serial dilution of synthetic *ESR1* E380Q stock oligonucleotide in a background of WT normal human DNA (*Taq*Man Control Genomic DNA) where the total DNA content of each ddPCR reaction was 20 ng and “wild-type double” was 40 ng. **b** The box plots of *ESR1* E380Q and *ESR1* WT detected in each input DNA. **c**, **d** The fluorescent signal (**C:** *ESR1* E380Q, **d**: *ESR1* WT) for each droplet is plotted on the y-axis for each dilution, which is separated by a dotted yellow line, with input DNA indicated **b**. The positive droplet fluorescence threshold is indicated by the magenta line. Blue dots represent FAM-labeled *ESR1* E380Q mutant DNA (**C**), green dots represent HEX-labeled WT DNA (**d**), and black dots are droplets with no DNA incorporated. Each droplet is cumulatively counted as an ‘Event Number’ for the ddPCR experiments analyzed in tandem, and plotted along the x-axis. Abbreviations; WT, wild-type; ddPCR, droplet digital polymerase chain reaction

Comparative ddPCR analysis of a dilution series of the indicated synthetic *ESR1* E380Q oligonucleotide is presented in Fig. 2a. We used serial dilutions of the *ESR1* E380Q molecule and analyzed them by ddPCR. A mixture of recombinant *ESR1* E380Q and WT *ESR1* was plotted against the different fractional concentrations from 32 to 0 copies/μL. The MAF of *ESR1* E380Q was maintained at more than two droplets. Therefore, a mutation was only considered to be present if more than two positive droplets were detected. In addition, we performed dilution experiments where the *ESR1* E380Q oligonucleotide was diluted in a background of WT normal human DNA (Fig. 2b-d). The dilution experiments were prepared by 1.3-fold serial dilution of synthetic *ESR1* E380Q stock oligonucleotide in a background of WT normal human DNA (*Taq*Man Control Genomic DNA) where the total DNA content of the ddPCR reaction was 20 ng and “WT double” was 40 ng. We confirmed that this assay was able to detect the *ESR1* E380Q molecule in a background of WT normal human DNA with the lowest concentration and was not able to detect any false-positives in the WT normal human DNA.

### The frequencies of *ESR1* mutations in primary and metastatic breast cancer patients

We screened nine PBC and 57 MBC tumors to detect *ESR1* E380Q and the other *ESR1* LBD mutations (Y537S, Y537N, Y537C, and D538G). The rates of *ESR1* mutations in primary and metastatic tumors from BC patients are shown in Table 2. We detected *ESR1* mutations in 21% (12/57) of MBCs, but could not detect them in primary breast tumors. The E380Q *ESR1* mutation was found in 16% (2/12) of *ESR1* mutant MBC patients, with a MAF of 21.7% (Case M43) and 2.69% (Case M50), respectively. Interestingly, two patients with the E380Q *ESR1* mutation had multiple metastatic sites. Other *ESR1* LBD mutations were five (41.6%) of Y537S, and four each (33.3%) of D538G, Y537N, and Y537C, in 12 *ESR1* mutant BC patients. We observed five cases with multiple *ESR1* LBD mutations in the same tumor: three had double *ESR1* mutations; Y537S/E380Q, Y37S/Y537C, and Y537S/D538G, and two had triple *ESR1* mutations; Y537S/Y537N/D538G.

**Table 2:** The number of *ESR1* mutations in primary and metastatic tumors from breast cancer patients

Samples	*N*	*ESR1* mutations					Patients with *ESR1* mutation	Rate of *ESR1* mutation
		E380Q	Y537S	Y537N	Y537C	D538G		
Primary	9	0	0	0	0	0	0	0
Metastasis								
Single site	24	0	2	2	2	3	6^a^	25% (6/24)
Multiple sites	33	2	3	2	2	1	6^b^	18.2% (6/33)

^a^Two patients had polyclonal *ESR1* mutations, Y537S/Y537N/D538G and Y537N/D538G

^b^Three patients had polyclonal *ESR1* mutations, Y537S/Y537N/D538G, Y537S/Y537C, and Y537S/E380Q

### Clinical course in patients with confirmed *ESR1* E380Q

Table 3 summarizes clinical characteristics and endocrine treatment history of patients with the E380Q *ESR1* mutation identified in ttDNA. Case M43 had metachronous bilateral BC. Clinical recurrence was detected at 16 months during adjuvant ET. She had previously received systemic treatment with five different therapies, not including ET, before MBC resection. A resected breast tumor had the E380Q *ESR1* mutation beyond the selected cut-off level. Hormonal therapy was administered in the adjuvant setting only and was not effective in this case. In case M50, ipsilateral breast tumor recurrence was detected at 72 months after primary surgery. This patient had received eight systemic therapies including four ETs before cervical lymph node biopsy. A cervical lymph node metastasis showed double *ESR1* mutations, the Y537S and the E380Q *ESR1* mutation, with a MAF of 12.8 and 2.69%, respectively. Concerning the effect of ET, some ETs were initially effective, but, eventually, all ETs including AI became ineffective in this case. There were an insufficient number of samples to formally analyze a predicted association between the E380Q *ESR1* mutation and patient prognosis.

**Table 3:** Clinical characteristics and endocrine treatment history in patients with confirmed *ESR1* E380Q mutant ttDNA

Clinical Characteristics								Before mutation analysis				After mutation analysis			
								CT	ET			CT	ET		
ID	Metastatic ER (%)	Detected *ESR1* mut	*ESR1* mut MAFs (%)	Stage at diagnosis	Adjuvant ET	Adjuvant ET Duration (Months)	DFI (Months)	No. of therapies	No. of therapies	Cumulative Exposure (Months)	Type of ET	No. of therapies	No. of therapies	Cumulative Exposure (Months)	Type of ET
M43 ^a^	70	E380Q	21.7	I	SERM	16	16	5	Adjuvant only			No	No	No	No
M50 ^a^	80	Y537S/ E380Q	12.8/2.69	I	LHRHa, SERM	48	72	4	4	40	LHRHa, SERM, AI	1	1	2	AI

Abbreviations; *ttDNA* tumor tissue DNA, *CT* chemotherapy, *ET* endocrine therapy, *ER* estrogen receptor, *mut* mutation, *MAF* mutant allele frequency, *SERM* selective estrogen receptor modulator, *LHRHa* luteinizing hormone-releasing hormone agonist, *DFI*, disease free interval, *No*. number, *AI* aromatase inhibitor

^a^Both patients were deceased

### Plasma cfDNA analysis

We screened the plasma cfDNA of nine PBC and 35 MBC patients to detect the E380Q *ESR1* mutation and the other *ESR1* LBD mutations (Y537S, Y537N, Y537C, and D538G). Plasma was collected at more than two points of the clinical course in nine PBC and 13 MBC patients (two blood draws in 10 patients and three blood draws in three patients) and we were able to investigate the change of *ESR1* LBD mutations in plasma cfDNA under treatment (Fig. 1). A total of six (46.2%) of 13 MBC patients showed increases in cfDNA *ESR1* LBD mutations under treatment and those were a useful tool, providing relevant predictive information as described previously [12]. However, the E380Q *ESR1* mutation was not identified in either the 1st blood samples or the serial blood samples under treatment in either the PBC group or the MBC group. Additionally, the E380Q *ESR1* mutation was not detected in plasma cfDNA even in two cases in which the E380Q *ESR1* mutation was identified in ttDNA: in Case M43 no plasma was collected and the status of *ESR1* mutation in plasma cfDNA was therefore unidentified. In Case M50 plasma was collected and cfDNA was analyzed, but did not have the E380Q *ESR1* mutation probably because the E380Q *ESR1* mutation in ttDNA was in a subpopulation of cancer cells.

## Discussion

In this study, we investigated the frequencies of the E380Q *ESR1* mutation in comparison with the other *ESR1* LBD mutations, Y537S, Y537N, Y537C, and D538G in tumor tissue and plasma DNA. In Vitro, Toy and colleagues showed differences in the ligand-independent activity among *ESR1* LBD mutations [6]. More recently, they also found tumors driven by D538G, E380Q or S463P were effectively inhibited by fulvestrant, but, Y537S mutants were not fully inhibited by fulvestrant despite dosing to higher levels than are achieved in the hospital [24]. Therefore, identification of the frequency and characteristics of each *ESR1* LBD mutation will deepen our knowledge and understanding of acquired ET resistance.

The raw data of E380Q *ESR1* mutation is shown in Additional file 1: Table S1. The measurement results of two experiments were processed to one value with QuantaSoft^™^ software (Bio-Rad Laboratories). Four early representative studies on *ESR1* mutations in ttDNA reported a total of one (2.5%) of E380Q in comparison with a total of 14 (35.8%) of D538G, 10 (25.6%) of Y537S, four (10.2%) of Y537N, three (7.6%) of Y537C, and seven other *ESR1* mutations among a total of 39 *ESR1* LBD mutation-positive ER-positive MBC patients [5–8]. More recently, deep sequencing of 929 breast tumor biopsies (including ER-positive, HER2-positive and ER-negative tumors) indicated 95 patients (10.2%) having somatic mutations in *ESR1*, which consisted of 20 (21.1%) of E380Q, 34 (35.8%) of D538G in comparison with 13 (13.7%) of Y537S, 6 (6.3%) of Y537C, and 5 (5.3%) of Y537N [24]. Meanwhile, in the recent clinical trials for plasma cfDNA of ET resistance MBC patients, E380Q *ESR1* mutation was found in 26% (15/57) [18] and 24.2% (22/91) of *ESR1* mutant plasma samples whose frequency was more than that of one of the major *ESR1* LBD mutations, Y537N [19].

The frequency of the E380Q *ESR1* mutation in our study seems to be rare among *ESR1* LBD mutations. We found a total of two (16.6%) E380Q *ESR1* mutation out of 12 MBC with *ESR1* LBD mutations and we did not find the E380Q *ESR1* mutation in plasma cfDNA (Table 2). Plasma cfDNA has the possibility to integrate *ESR1* mutations from distinct populations of cells which are caused by inter- and/or intra-tumoral heterogeneity [11, 25]. However, the E380Q *ESR1* mutation was not identified in any of our 69 analyzed plasma cfDNA samples. In another small cohort of HR-positive Japanese MBC patients, whole exon sequencing of the *ESR1* gene using NGS did not identify E380Q *ESR1* mutation in their recurrent tumor samples and plasma samples [26].

Identifying associations between the status of the E380Q *ESR1* mutation and response to ET will help to use ET more effectively. Li and colleagues detected the E380Q *ESR1* mutation in an ER-positive patient-derived xenograft that reacted to tamoxifen, but was resistant to AI*.* [15]. De Mattos-Arruda and colleagues reported that the MAF of the E380Q *ESR1* mutation in plasma cfDNA increased from 46 to 58% under disease progression [16]. However, in this study, there were an insufficient number of samples to formally analyze a predicted association between the E380Q *ESR1* mutation and the patient’s prognosis. The present study has limitations. This was a retrospective and single-institute study. Since this was a selected mutation-based study, not all the *ESR1* LBD mutations were investigated. The number of patients with the E380Q *ESR1* mutation was small due to the selection criteria. Although the appearance of *ESR1* LBD mutations is closely associated with medical history of ET, this studied population is heterogeneously treated and we could not investigate whether or not the presence of *ESR1* LBD mutations is dependent on specific hormone therapies.

## Conclusions

We demonstrated the presence of a distinct population of *ESR1* LBD mutations (E380Q, Y537S, Y537N, Y537C, and D538G) in metastatic tissue and plasma using ddPCR assay. The identification of recurrent *ESR1* mutations in metastatic ER-positive BCs may provide the basis of understanding ET resistance mechanisms, which may contribute to the development of new molecular targeted therapy.

## Additional file

Additional file 1: Table S1. The raw data of E380Q *ESR1*mutation. Abbreviation: Conc., Concentration. (XLSX 15 kb)

## Abbreviations

AI: Aromatase inhibitor
BC: Breast cancer
cfDNA: Cell-free DNA
ddPCR: Droplet digital polymerase chain reaction
ER: Estrogen receptor
ET: Endocrine therapy
HER2: Human epidermal growth factor receptor 2
HR: Hormone receptor
LBD: Ligand-binding domain
LMD: Laser microdissection
MAF: Mutant allele frequency
MBC: Metastatic breast cancer
mTOR: Mammalian target of rapamycin
NGS: Next-generation sequencing
PBC: Primary breast cancer
PgR: Progesterone receptor
ttDNA: Tumor tissue DNA
WT: Wild-type
